# Evaluation of the anti-angiogenic action of melatonin in breast cancer

**DOI:** 10.1186/1753-6561-7-S2-P11

**Published:** 2013-04-04

**Authors:** Bruna V Jardim, Lívia C Ferreira, Thaiz F Borin, Marina G Moschetta, Gabriela B Gelaleti, Juliana R Lopes, Larissa B Maschio, Camila Leonel, Naiane N Gonçalves, Gustavo R Martins, Debora APC Zuccari

**Affiliations:** 1 Department of Biology, Universidade Estadual Paulista (UNESP/IBILCE), São José do Rio Preto, SP, Brazil; 2 Laboratório de Investigação Molecular do Câncer, Faculdade de Medicina de São José doRio Preto (FAMERP), São José do Rio Preto, SP, Brazil; 3 Departament of Molecular Biology, Faculdade de Medicina de São José do Rio Preto (FAMERP), São José do Rio Preto, SP, Brazil

## Background

Once a tumor lesion exceeds a few millimeters in diameter, hypoxia triggers a cascade of events to allow angiogenesis and tumor progression. As angiogenesis is essential for tumor growth and metastasis, controlling tumor-associated angiogenesis is a promising tactic in limiting cancer progression. Melatonin has been suggested to inhibit angiogenesis in cancers, although this effect has not been described in breast cancer. We evaluated the effects of melatonin treatment on angiogenesis in breast cancer.

## Materials and methods

MDA-MB-231 breast cancer cell line was cultured in DMEM high glucose at 37°C in 5% CO_2_. Cells received CoCl_2_ to mimic hypoxia and were then treated with melatonin (1mM). Cell viability was measured by MTT assay, and protein and gene expression were assessed by immunocytochemistry and real time PCR, respectively. We performed an *in vivo* study where cells were implanted in the mammary gland of athymic nude mice. Mice were treated with 1mg of melatonin or vehicle daily, administered intraperitoneally 1 hour before room lighting was switched off. Tumors were measured weekly with a digital caliper and angiogenic proteins were evaluated in mammary tumor tissues.

## Results

Melatonin *in vitro* treatment was able to significantly decrease cell viability and protein expressionof the hypoxia inducible factor 1 alpha (HIF1α), under hypoxic conditions. Furthermore, the anti-angiogenic action of melatonin was tested with breast cancer xenografts in nude mice.

## Conclusion

This is the first study to show that melatonin effectively acts against angiogenesis in breast tumors, suggesting that melatonin may have potential therapeutic applications in this disease.

## Financial support

FAPESP.

